# Annexin A1: A Bane or a Boon in Cancer? A Systematic Review

**DOI:** 10.3390/molecules25163700

**Published:** 2020-08-14

**Authors:** Thanusha Ganesan, Ajantha Sinniah, Zaridatul Aini Ibrahim, Zamri Chik, Mohammed Abdullah Alshawsh

**Affiliations:** Department of Pharmacology, Faculty of Medicine, University of Malaya, Kuala Lumpur 50603, Malaysia; thanushaganesan@gmail.com (T.G.); zaridatulaini@um.edu.my (Z.A.I.); zamrichik@ummc.edu.my (Z.C.); alshaweshmam@um.edu.my (M.A.A.)

**Keywords:** Annexin A1, lipocortin 1, cancer, gene expression, systematic review

## Abstract

Annexin A1 has been extensively investigated as an anti-inflammatory protein, but its role in different types of cancer has not been consolidated in a single systematic review to date. Thus, the aim of this paper is to systematically review and critically analyse 18 studies (in-vivo and in-vitro) to consolidate, in a concerted manner, all the information on differential expression of Annexin A1 in different types of cancer and the role this protein plays in tumorigenesis. Pubmed, Scopus, Web of Science, and ScienceDirect were used for the literature search and the keywords used are “annexin A1,” “lipocortin 1,” “cancer,” “malignancy,” “neoplasm,” “neoplasia,” and “tumor.” A total of 1128 articles were retrieved by implementing a standard search strategy subjected to meticulous screening processes and 442 articles were selected for full article screening. A total of 18 articles that adhered to the inclusion criteria were included in the systematic review and these articles possessed low to moderate bias. These studies showed a strong correlation between Annexin A1 expression and cancer progression via modulation of various cancer-associated pathways. Differential expression of Annexin A1 is shown to play a role in cellular proliferation, metastasis, lymphatic invasion, and development of resistance to anti-cancer treatment. Meta-analysis in the future may provide a statistically driven association between Annexin A1 expression and malignancy progression.

## 1. Introduction

### 1.1. Annexin A1 (Anx-A1) at a Glance

In recent years, there has been an exponential interest in the role of Annexin A1 (Anx-A1) in cancer due to the increasing number of studies investigating the effect of this endogenous protein in different types of cancer. The appreciation that is growing on Anx-A1 can be attributed to the emerging evidence regarding the potential of Anx-A1 to be a diagnostic marker as well as a therapeutic target in cancer. 

Anx-A1 is a calcium and phospholipid-binding protein with a molecular weight of 37 kDa formed by 346 amino acids and it is the first member of the Annexin superfamily to be discovered [1]. A core domain with four repeating conserved 60–70 amino acid motifs is an intrinsic feature of the annexin family. The core domain is attached to an *N*-terminus (distinctive to each annexin), which is responsible for the regulatory mechanisms of Anx-A1 [2]. Another intrinsic feature of Anx-A1 is its ability to alter its conformation upon binding to positively charged calcium ions [3]. The structural alteration of Anx-A1 as a result of interaction between the core domain and phospholipids implicates the biology of the protein, particularly its interaction with the potential receptors [1]. The interaction results in multiple biological functions of the Anx-A1 protein such as anti-inflammation, pro-apoptosis, induction of cytokine, anti-pyretic, regulation of junctions of blood brain barriers, regulation of angiogenesis, and the discoveries that ensue [4]. The diversified biological functions of this protein have attracted the attention of scientists to research its role in different diseases including cancer. 

In order for Anx-A1 to exert its biological functions, it has to be externalized by its cellular sources. However, an insufficient signalling peptide causes secretion via traditional pathway impossible [4]. Hence, the protein needs to be induced by glucocorticoid to catalyse externalisation and localisation of Anx-A1. Nevertheless, it likely is not the sole mechanism by which externalisation of Anx-A1 happens in our body [5].

### 1.2. Anx-A1 in Cancer

Alterations in expression of Anx-A1 and changes in its localization have been associated with the progression of different malignancies [4]. Indeed, increased levels of Anx-A1 have been reported in lung [6,7], hepatocellular [8], colorectal [9,10,11], pancreatic [12], and skin cancer [13]. Meanwhile, prostate [14,15,16], cervical [17], nasopharyngeal [18,19], lymphoma [20], oral squamous cell carcinoma [19,21], and larynx cancer [22] show down-regulation of Anx-A1 expression. Increased expression of Anx-A1 is often related to disease severity, advanced tumour stage, and metastasising and invading potential tumour cells [4]. 

A study by Xia et al. (2002) has reported a positive correlation between Anx-A1 levels and the differentiation of oesophageal cancer whereby well-differentiated cells have higher expression of Anx-A1 and vice versa [23]. In another study, carcinogenesis and enhanced tumour aggressiveness in prostate cancer due to an elevated level of interleukin-6 (IL-6) is linked to the down-regulation of the Anx-A1 level [24]. An intriguing fact about breast cancer is that conflicting data on expression of Anx-A1 were reported, as there are findings on overexpression of Anx-A1 in tumour ducts [25] and a more recent finding revealed lower expression of Anx-A1 in in-situ and invasive ductal carcinoma in comparison with normal epithelium [26].

These discrepancies indicate a ‘yin and yang’ effect of Anx-A1 in cancer, which enhances the understanding of the role of the protein in depth. This can further unravel the potential of Anx-A1 to be developed as a diagnostic marker and a pharmacological target in the treatment of cancer. Hence, this systematic review aims to capture the differential expression of Anx-A1 in 11 types of cancer and relate the respective roles of Anx-A1 in cancer to the varied expression.

## 2. Methods 

The design of the study was devised based on the Preferred Reporting Items for Systematic Reviews and Meta-Analysis (PRISMA) and the Cochrane Collaboration [27,28]. All the primary studies included in this systematic review are preclinical studies related to Anx-A1 and cancer and no Institutional Review Board or patients’ informed consent were required considering the nature of the included studies in this review, which comprise in-vitro and in-vivo studies. Two independent researchers were involved in different stages of the review such as literature search, decision on inclusion and exclusion criteria, risk of bias assessment, and data extraction in order to eliminate any bias in the assessment. Registration of the systematic review protocol was done with the international prospective register of systematic reviews database “PROSPERO” in March 2018 and the registration number is CRD42018090706.

### 2.1. Search Strategy 

A systematic search strategy was used to retrieve the most relevant articles from PubMed, Scopus, Web of Science, and ScienceDirect databases. In PubMed, Medical subject heading (MeSH) terms were initially used to derive synonymous keywords to devise a search question. The keywords and Boolean operators included in the search were “annexin A1 OR lipocortin 1 AND cancer OR malignancy OR neoplasm OR neoplasia OR tumor.” The same search strategy was applied to the other three databases as well. Grey literature including Google Scholar and Proquest were searched to ensure all the relevant studies have been retrieved including unpublished data and thesis. The search period was limited to 31 December, 2017 and only English journals were included for the review. 

### 2.2. Study Selection and Eligibility Criteria 

**Figure 1:**
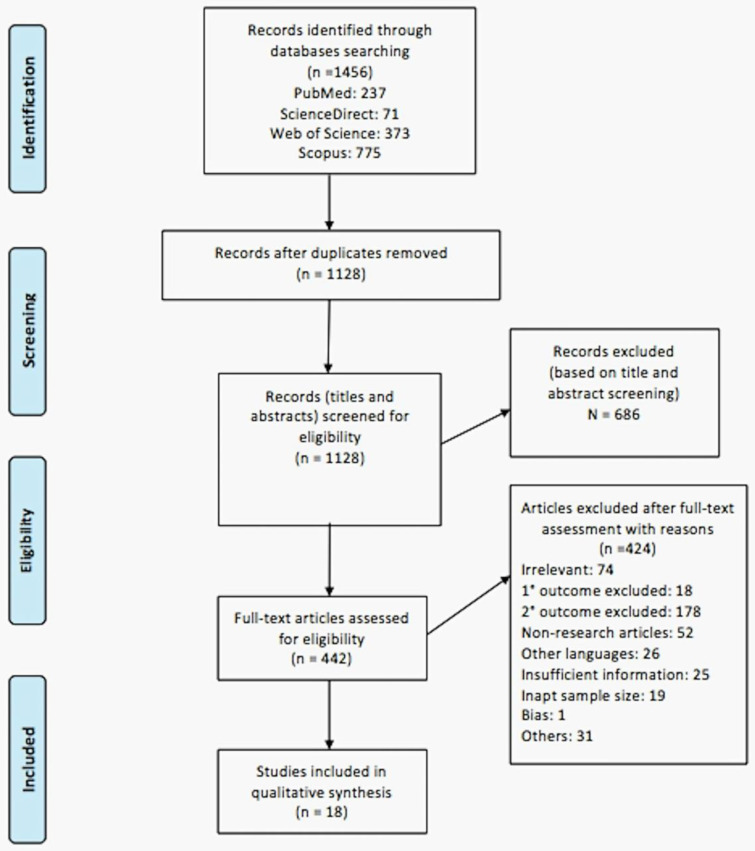
PRISMA flow diagram of study selection process.

Using the search strategy devised, 1128 articles (after removing duplication) were retrieved from the four databases and, upon screening the titles and abstracts, 442 relevant articles were selected for full text assessment based on the inclusion and exclusion criteria pre-determined by the researchers. Upon assessment of full articles, only 18 articles were included for final qualitative analysis in this systematic review (Figure 1). 

Studies included adhered to the following inclusion criteria: (1) In-vivo and in-vitro pre-clinical studies on cancer (all types) and Anx-A1, (2) studies that included gene and protein expression and role of Anx-A1 in different types of cancer, and (3) studies with or without interventions. The exclusion criteria were: (1) In-vivo studies investigating other disease conditions, (2) interventions unrelated to cancer or Anx-A1, (3) studies with insufficient data outcome, poorly elaborated methodologies, bias outcome, and insufficient sample size, and (4) journal articles in languages other than English. The studies included in this systematic review comprised of both primary and secondary outcomes (as per PROSPERO registration) and studies missing either of the outcomes were excluded. 

### 2.3. Data Extraction

The data were extracted using a pre-devised table, which contains the following information: type of study, type of cancer, type of cell line/animal model, treatment/intervention details, primary outcome (expression of Anx-A1), secondary outcome (role of Anx-A1), and limitations of the study. The information was then summarised concisely in Table 1. Any discrepancy in data extracted by two independent researchers was resolved by a third researcher to avoid any bias in acquisition of important data. 

### 2.4. Risk of Bias Assessment

Risk of bias and quality assessment for the included studies in this systematic review was divided into two parts (in-vivo and in-vitro) and different risk of bias tools were employed to assess the bias in the studies selected. SYRCLE’s tool was used to assess the quality of animal studies and the parameters assessed were selection bias, performance bias, detection bias, attrition bias, reporting bias, and other sources of bias [29] (Table 2). On the other hand, there is no standard risk of bias tool to assess the in-vitro studies published thus far. Hence, as per PROSPERO team’s suggestion, we modified the parameters of existing OHAT assessment tool to fit the quality analysis of in vitro studies included in our review [30]. The parameters assessed under the OHAT tool were similar to SYRCLE’s tool with the specific criteria assessed for each parameter differing slightly to suit the nature of the in vitro study being analysed (Table 3). 

## 3. Results and Discussion

Differential expression of Anx-A1 in various types of cancer and corresponding biological roles, which can either be pro-cancer or anti-cancer has captured the interests of scientists worldwide. Hence, this systematic review was carried out to critically analyse the role of this protein in cancer and to explore possibilities to develop potential therapeutic targets for the disease.

### 3.1. Studies Screening and Selection

After abstracts’ and titles’ screening processes and removal of duplication, 442 articles were included for a full text assessment (Figure 1). Upon completion of assessment of an individual article, the decision to include or exclude the articles was done based on pre-determined inclusion and exclusion criteria. The articles that were not included were given reasons for their exclusion. The reasons for exclusion are irrelevance (*n* = 74), exclusion of primary outcome (*n* = 18), exclusion of secondary outcome (*n* = 178), non-research articles (*n* = 52), articles in different languages (*n* = 26), insufficient information (*n* = 25), inadequate sample size (*n* = 19), bias (*n* = 1), and other reasons (*n* = 31), which include insignificant results, irretrievable papers, and poorly structured/written papers. Lastly, eighteen articles that qualified the inclusion criteria were included in this review for qualitative synthesis. The decision to not proceed with meta-analysis was made after analysing the articles and deducing that pooling of homogenous data is not possible from the selected studies. 

### 3.2. Quality of Included Studies

The risk of bias assessment, which was done following inclusion of selected studies in the review, was aimed at ensuring that the outcome of the studies are not largely affected by the prevailing bias in the primary studies. Generally, regardless of the type of pre-clinical studies (let it be in-vitro or in-vivo), each domain of bias was assessed and categorised to possess high, low, or an uncertain level of bias. Should an article have a high level of bias (more than four domains recorded high), the article was excluded to prevent inclusion of lopsided findings. The journals of articles included were also checked for predatory journals using Beall’s list in order to ensure studies included are retrieved from credible sources. All these measures are intended to ensure the outcomes extracted and consolidated for the review are highly reliable and credible. 

Generally, the levels of bias for the 18 in vitro studies included in this systematic review ranged from ‘probably low’ to ‘probably high’ with the maximum number of domains with ‘probably high’ level of bias reaching three. On the other hand, for in-vivo experiments, none of the studies recorded a high level of bias with the majority of the studies having a low and an uncertain level of bias for all the domains (refer to Table 2 and Table 3). 

### 3.3. Expression and Localisation of Anx-A1

It has been demonstrated that Anx-A1 is differentially expressed in different types of cancer. Anx-A1 is up-regulated in the brain tumour [38], lung cancer [39,46], and melanoma [42] while the expression is reduced in breast cancer [33] and cervical cancer [17]. Differential expression of Anx-A1 is not only limited to different types of cancer but also applies to different types of cell lines within the same type of cancer (Table 1). For instance, higher expression of Anx-A1 was found in basal-like breast cancer (BLBC) cells than luminal-like breast cancer (LLBC) cells in breast tumours [33] while, in pancreatic cancer, PaCa-2 and PANC-1 cell lines express different levels of cleaved (33 kDa) and full length of Anx-A1 (37 kDa), respectively, despite the cells originating from the same type of malignancy [41]. 

Variation in Anx-A1 expression can also be attributed to different stages of cancer, which have varying invasive properties and differentiation levels of the cell lines. In cervical cancer, significant down-regulation of Anx-A1 was observed in poorly differentiated cancer cells [17] and pancreatic ductal adenocarcinoma also shares a similar pattern of association between Anx-A1 expression and cellular differentiation [45]. On the contrary, lung cancer illustrates higher expression of Anx-A1 in poorly differentiated lung tissues and an increasing trend of Anx-A1 expression is also related to increasing tumour stages [39,46]. Some studies have reported linear correlation between expression of Anx-A1 and invasive potential and metastasising ability of lung cancer [39,46] and breast cancer [40]. Nevertheless, contradicting results were reported for breast cancer [36] as well as for nasopharyngeal carcinoma [43] in which declining expression of the protein was correlated with increasing metastatic ability of the tumours.

Anx-A1 also shows differential cellular localisation, depending on cancer types, which could have a critical role in tumorigenesis. The three most commonly explained localisations of Anx-A1 in cancer are cytoplasmic, nucleic, and attached either closely or loosely to the plasma membrane. In one study done on human tissue samples collected from melanoma patients, it was shown that Anx-A1 is localised mostly in the cytoplasm [42]. Another investigation on two different pancreatic cancer cell lines revealed that the localisation of Anx-A1 was in the membrane and cytoplasm for PaCa-2 pancreatic carcinoma cells while, in PANC-1 cells, it is localised to the membrane and nucleus but not to cytosol [41].

Differential expression of Anx-A1, its association with multiple aspects of cancer and variations in localisations of the protein collectively provide an insight of the important role that this protein is playing in the progression or inhibition of malignancy. The discrepancies disclose the possibility of Anx-A1 to be developed either into a prognostic or a diagnostic marker.

### 3.4. Roles of Anx-A1 in Cancer

Roles played by Anx-A1 in cancer are multi-faceted since it impinges on molecular and genetic changes that consequently modulate multiple pathways linking tumorigenesis. Its role in cancer includes regulation of cellular proliferation, metastasis, lymphatic invasion, development of resistance to anti-cancer treatment, and modulation of cancer-related signalling pathways.

Two studies reported Anx-A1’s involvement in the development of resistance to anti-cancer treatment. However, the correlation between the expression of Anx-A1 and drug resistance were contradictory [31,32]. In breast cancer, higher expression of Anx-A1 contributed to depletion of sensitivity to anti-cancer drugs [31], whereas, in chronic myeloid leukemia (CML), drug resistance was associated with lower expression of Anx-A1 [33]. Studies on esophageal cancer [32] and cervical cancer [17] elucidate the possible role of Anx-A1 as a prognostic marker and a tumour suppressor protein. In addition, a few other studies investigated the function of the steroid-induced protein in regulating cellular proliferation, cell cycle arrest, and colony stimulation in a few tumour types. Up-regulation of Anx-A1 inhibits cellular proliferation, colony formation, and leads to cell cycle arrest in breast cancer [33] and nasopharyngeal carcinoma [43]. Knocking down Anx-A1 in the breast cancer cell line and the animal model increases cellular proliferation and speeds up tumour growth and obliterates cell cycle arrest, which proves an anti-proliferative effect of Anx-A1 [33]. On the contrary, in human glioblastoma, knockdown of the same protein is needed to elicit opposite effects including inhibition of colony stimulation of the cells and infiltration of leucocytes showing that Anx-A1 can also act as a pro-cancer protein [38].

Apart from different roles explained above, metastasis is one aspect of cancer that is largely linked to Anx-A1. The role that Anx-A1 plays in metastasis is inevitable as the effect is prevalent in almost all types of cancers studied. There are conflicting findings on the role of Anx-A1 in metastasis of breast cancer. De Grauuw et al. (2010) explained that depleting Anx-A1 reduces the migratory distance and speed, induces cell-cell junction formation, and down-regulates the TGFβ/SMAD pathway, which is vital for epithelial-mesenchymal transition (EMT) in metastasis [35]. In contrast, transfection of Anx-A1 into MDA-MB-231, which is the estrogen-independent breast cancer cell line, was found to reverse the EMT phenotype back to epithelial and the effect of Anx-A1 in metastasis deemed independent of the TGFβ/SMAD pathway [36]. Anx-A1 seems to inhibit metastasis by regulating various pathways and modulators, which include down-regulation of the TGFβ/SMAD pathway [35], down-regulation of the IKK complex, and NF-κB pathway [37], down-regulation of MMP-9, and its proteolytic activity [40,45], down-regulation of the MMP-2 protein [42], inhibition of the mTOR–S6 pathway, and increase of AMPKα phosphorylation [44]. Role of Anx-A1 in metastasis of pancreatic cancer was studied by agonizing and antagonizing formyl peptide receptor (FPR) 1 and 2, which are cognate receptors for Anx-A1 [41]. Agonizing FPR with Ac2-26 increases the migration rate, invasion speed, and contradictory results were observed in the presence of the FPR antagonist Boc-1 [41].

Whether up-regulation or depletion of Anx-A1 translates these biological changes depends on the type of cancer investigated, given that implication of Anx-A1 in tumorigenesis is cell-specific. In other words, the effect can vary between different cell types within the same type of tumour.

### 3.5. Anx-A1 and Cancer-Related Mechanisms

**Figure 2:**
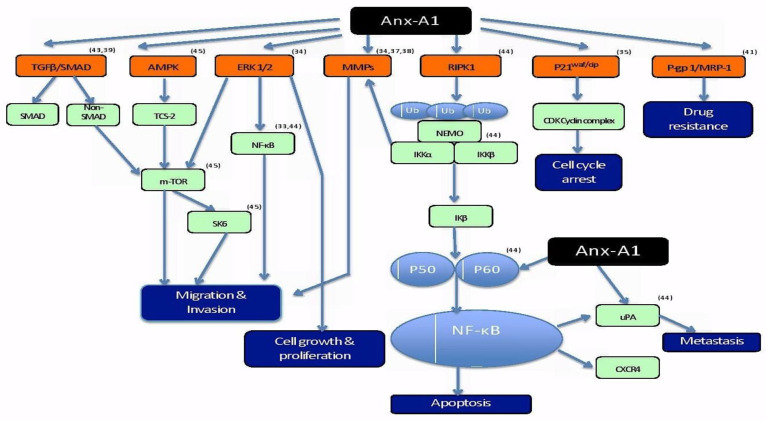
Schematic representation of differential roles of Anx-A1 in various signaling pathways contributing to the pathophysiology of cancer [47,48,49,50].

Role of Anx-A1 in cancer is distinct to not just different cancer types but to different types of cells within a cancer. These extended roles of Anx-A1 in cancer is evidence of Anx-A1’s involvement in multiple pathways driving the pathogenesis of a tumour. The fact that the mechanism of action of Anx-A1 is distinctive to each type of cancer proposes a challenge in generalizing possible pathways modulated by the protein for all types of cancer. Hence, in an attempt to amalgamate the information on the modulation of different pathways by Anx-A1, a schematic diagram has been drawn to give a general overview of cancer-associated pathways affected by this protein (Figure 2).

Anx-A1 has differing roles in different cancer-related pathways that lead to multiple hallmark characteristics of the disease. Shown in the diagram above are the corresponding pathways to differentially expressed Anx-A1 in different types of cancer and the proteins/molecules involved in regulating the involved pathways from the studies included in this systematic review.

Based on the studies included, the pathways that are affected by Anx-A1 include TGFβ/SMAD, AMPK, ERK1/2, MMP, RIPK1, P21^waf/cip^, and P-gp1/MRP-1 pathways. Anx-A1 regulates multiple proteins that can directly or indirectly stimulate or inhibit a particular pathway contributing to the pathogenesis of cancer. Regulation of the multiple pathways leads to different hallmark characteristics of cancer that are fundamental for cancer progression. Migration/invasion is driven by TGFβ/SMAD [35,36], AMPK [44], ERK ½ [33], and MMP [40,42,45] pathways. According to Ang et al. (2009), ERK 1/2 pathway is also involved in regulating cell growth and proliferation of malignant cells [33]. Apoptosis, which is another key feature futile in cancer, is regulated by the RIPK1 pathway that, in turn, modulates NF-κB, which is a crucial protein for transcription of DNA, cytokine production, and survival of cells. Inactivation of NF-κB by Anx-A1 either directly or indirectly inhibits downstream signaling of RIPK1 pathway, disrupts apoptosis, and, consequently, leads to cancer progression [37]. The mechanism of the cell cycle arrest is often dysfunctional in cancer in order to enable unrestricted cellular proliferation and blocking of Anx-A1 can potentially inhibit P21^waf/cip^ to inactivate the cell cycle arrest [33]. In addition, overexpression of Anx-A1 contributes to one of the biggest challenges in treating cancer, which is drug resistance. It has been hypothesized that overexpression of Anx-A1 causes drug resistance via aggregation and exocytosis of vesicles containing drugs [31,51]. These different pathways may have more extensive cross-roles. However, the diagram only captures the outcomes from the articles included in the systematic review with a purpose of drawing a summary on the impact of Anx-A1 on cancer-related pathways.

More extensive studies will be needed to find (a) common pathway(s) regulated by Anx-A1 in the most common types of cancers in order to understand the biology of this protein in depth.

## 4. Conclusions

This systematic review demonstrates diverse biological roles that Anx-A1 implicates in different types of cancer including proliferation, metastasis, survival, and resistance. For the first time, we discussed the dual and spatial expression of Anx-A1 in most types of cancer both in-vitro and in-vivo and how that contributes to the development or inhibition of respective cancer types. However, contradictory evidence presented in the studies further complicate understanding the dynamics of Anx-A1. In order to develop a more comprehensive and coherent understanding on how Anx-A1 interplays in cancer pathways, future studies should focus on more diverse tumour models with a specific focus on important parameters. For instance, manipulation of metastasis-related genes in different types of cancer to study the role of the Anx-A1 in the spread of tumours would provide a comprehensive insight on a particular role of protein in various cancers rather than one. A better understanding on biological roles of Anx-A1 can be utilized to exploit the development of therapeutic targets of Anx-A1, FPR, or signaling pathways related to the cancer.

## Figures and Tables

**Table 1:** Summary findings of the studies included in the systematic review.

Study	Type of Study/Study Design	Cancer Types	Primary Outcome Expression of Anx-A1	Secondary Outcome Role of Anx-A1 in Cancer
[31]	In vitro	Ovarian and breast cancer	Increased Anx-A1 expression in the drug-resistant cells (SKOV-3) compared to the drug sensitive (MCF-7) cells	Resistance towards anti-cancer treatment
[26]	In vitro	Breast cancer	Reduced Anx-A1 expression in ductal cell in situ (DCIS) and invasive breast tumour Primary tumour and metastatic cells showed significantly lower Anx-A1 expression than normal mammary cells Primary tumour showed significantly lower expression of Anx-A1 in comparison to the corresponding metastatic cells in the lymph nodes	Possible tumour suppressor activity of Anx-A1
[32]	In vitro	Esophageal and esophageal junction adenocarcinoma	61% (63 out of 104) of esophageal and esophagogastric junction adenocarcinomas showed negative or low expression of Anx-A1 39% (41 out of 104) of esophageal and esophagogastric junction adenocarcinomas recorded high expression of Anx-A1 High Anx-A1 expression correlated to poorer prognosis, higher tumour stage and distant metastasis	Prognosis marker to predict tumour recurrence and survival of patients
[17]	In vitro	Cervical cancer	The difference in Anx-A1 expression between normal cervical epithelium and cervical intraepithelial neoplasia (CIN) and invasive squamous cell carcinoma (ISCC) was significant The expression of Anx-A1 significantly decreased with tumour severity Low Anx-A1 correlated to poorer cellular differentiation of the tumour	Possible tumour suppressor protein and diagnostic marker
[33]	In vitro and in vivo	Breast cancer	Anx-A1 expression was negative in low-grade ductal carcinoma, ductal carcinoma in-situ (DCIS), and invasive ductal carcinoma Anx-A1 stained positive for myoepithelial and epithelial cells.	Cell proliferation and cell cycle arrest
[34]	In vitro	Chronic myeloid leukemia (CML)	In adriamycin sensitive K562 cells, high Anx-A1 was detected while in the resistant, K562/ADR cells, the expression was largely reduced.	Resistance towards anti-cancer treatment
[35]	In vitro	Breast cancer	Anx-A1 expression was higher in basal like breast cancer (BLBC) cell than luminal like breast cancer cell (LLBC)	Metastasis
[36]	In vitro and in vivo	Breast cancer	Anx-A1 expression was recorded high in a normal breast tissue sample while found downregulated in 20 invasive human breast carcinoma, excluding four benign breast tissue samples	Metastasis
[37]	In vitro and in vivo	Breast cancer	MCF-7 and T47D cells expressed lower levels of Anx-A1 while MDA-231 cells showed higher Anx-A1 level	Metastasis
[38]	In vitro and in vivo	Human glioblastoma	Anx-A1 was negative in normal brain tissue but upregulated in brain tumour tissue, higher grade gliomas (grade 3 and 4) recorded higher Anx-A1 expression	Cell proliferation, colony stimulation, infiltration of leucocytes, and survival
[39]	In vitro	Lung cancer	Anx-A1 was significantly upregulated in 44 of the 96 cancer tissues and downregulated in normal tissues (*p* < 0.05) mRNA levels of Anx-A1 were significantly increased in lung cancer tissues than the normal tissues (*p* = 0.02)	Metastasis and survival
[40]	In vitro	Breast cancer	MDA-MB-213 cells showed high Anx-A1 expression, while SKBr3 and T47D cells had low expression and the MCF-7 cell line was negative for the protein	Metastasis
[41]	In vitro	Pancreatic cancer	In PaCa-2 extracts, full length (37kDa) and cleaved (33kDa) forms of Anx-A1 were found in the membrane and cytosol but not in the nucleus PANC-1 did not express a cleaved form of Anx-A1 but expressed the full length in a small amount on the membrane and in the nucleus	Metastasis
[42]	In vitro and in vivo	Melanoma	Expression of Anx-A1 was found in 54/61 biopsies with the membrane as well as the cytoplasmic and nuclear compartments amongst which cytoplasmic localization was the highest (51/54)	Metastasis
[43]	In vitro	Nasopharyngeal carcinoma (NPC)	6-10B cells have notably increased expression of Anx-A1 compared to the metastatic 5-8F cell lines	Cell proliferation, cell cycle arrest, colony formation and metastasis
[44]	In vitro	Breast cancer	Higher endogenous Anx-A1 expression was identified in TNBC cell lines than the non-TNBC cells	Metastasis
[45]	In vitro	Pancreatic ductal adenocarcinoma (PDAC)	Positive Anx-A1 staining was mainly found in cytoplasm (with or without nucleus) Anx-A1 expression was significantly decreased in the tumour with a higher TNM stage (3-4) than lower stages (1-2)	Metastasis and survival
[46]	In vitro	Lung cancer	Lung cancer tissue showed higher expression of Anx-A1 than in normal lung tissue	Lymphatic invasion

**Table 2:** Risk of bias assessment for in-vitro studies using the modified OHAT tool.

Studies	Selection Bias		Performance Bias			Detection Bias		Attrition Bias	Reporting Bias	Others
	Randomization	Allocation Concealment	Experimental Conditions	Exposure Characterization	Blinding	Random Outcome Assessment	Blinding	Incomplete Outcome Data	Selective Outcome Reporting	Other Sources of Bias
[31]	PL	PL	DL	DL	PH	PL	PH	PL	PL	PH
[26]	PL	PL	PL	PL	PH	PL	PH	PL	PL	PL
[32]	PL	PL	DL	DL	PH	PL	PH	DL	PL	DL
[17]	PL	PL	PL	PL	PH	PL	PH	PL	PL	DL
[33]	PL	PL	DL	DL	PH	PL	PH	PL	DL	DL
[34]	PL	PL	DL	DL	PH	PL	PH	PL	PL	DL
[35]	PL	PL	DL	DL	PH	PL	PH	DL	DL	DL
[36]	PL	PL	DL	DL	PH	PL	PH	PL	PL	PL
[37]	PL	PL	DL	DL	PH	PL	PH	DL	DL	DL
[38]	PL	PL	PL	PL	PH	PL	PH	DL	PL	DL
[7]	PL	PL	DL	PL	PH	PL	PH	DL	PL	DL
[40]	PL	PL	DL	PL	PH	PL	PH	PL	PL	DL
[41]	PL	PL	DL	PL	PH	PL	PH	DL	PL	DL
[42]	PL	PL	DL	PL	PH	PL	PH	DL	PL	DL
[43]	PL	PL	DL	PL	PH	PL	PH	DL	PL	DL
[44]	PL	PL	DL	PL	PH	PL	PH	PL	PL	DL
[45]	PL	PL	PL	PL	PH	PL	PH	DL	PL	DL
[46]	PL	PL	PL	PL	PH	PL	PH	DL	PL	DL

PL: Probably low. DL: Definitely low. PH: Probably high. DH: Definitely high.

**Table 3:** Risk of bias assessment for in-vivo studies using SYRCLE’s tool.

Studies	Selection Bias			Performance Bias		Detection Bias		Attrition Bias	Reporting Bias	Others
	Random Sequence Generation	Baseline Characteristics	Allocation Concealment	Random Housing	Blinding	Random Outcome Assessment	Blinding	Incomplete Outcome Data	Selective Outcome Reporting	Other Sources of Bias
[33]	U	L	U	U	U	L	U	L	L	L
[36]	U	U	U	L	U	L	U	L	L	U
[37]	U	L	U	L	U	L	U	L	L	L
[38]	U	U	U	U	U	L	U	L	L	L
[42]	U	L	U	U	U	U	U	L	L	L

L: low risk of bias. H: high risk of bias. U: Uncertain.
